# Trap Characterization Techniques for GaN-Based HEMTs: A Critical Review

**DOI:** 10.3390/mi14112044

**Published:** 2023-10-31

**Authors:** Xiazhi Zou, Jiayi Yang, Qifeng Qiao, Xinbo Zou, Jiaxiang Chen, Yang Shi, Kailin Ren

**Affiliations:** 1 School of Microelectronics, Shanghai University, Shanghai 200444, China; xiazhizou@shu.edu.cn (X.Z.); 1142048504@shu.edu.cn (J.Y.); shiyang@shu.edu.cn (Y.S.); 2 Shanghai Key Laboratory of Chips and Systems for Intelligent Connected Vehicle, Shanghai University, Shanghai 200444, China; 3 Shanghai Industrial μTechnology Research Institute, Shanghai 201800, China; 4 School of Information Science and Technology, ShanghaiTech University, Shanghai 201210, China; zouxb@shanghaitech.edu.cn (X.Z.); chenjx2@shanghaitech.edu.cn (J.C.)

**Keywords:** GaN HEMT, trap, characterization methods, DLTS

## Abstract

Gallium nitride (GaN) high-electron-mobility transistors (HEMTs) have been considered promising candidates for power devices due to their superior advantages of high current density, high breakdown voltage, high power density, and high-frequency operations. However, the development of GaN HEMTs has been constrained by stability and reliability issues related to traps. In this article, the locations and energy levels of traps in GaN HEMTs are summarized. Moreover, the characterization techniques for bulk traps and interface traps, whose characteristics and scopes are included as well, are reviewed and highlighted. Finally, the challenges in trap characterization techniques for GaN-based HEMTs are discussed to provide insights into the reliability assessment of GaN-based HEMTs.

## 1. Introduction

Gallium nitride (GaN) high-electron-mobility transistors (HEMTs) possess superior features such as high breakdown voltage, high electron saturation drift velocity, and low ON-resistance, making them highly promising for power device applications [1,2]. Additionally, due to the polarization effect in GaN-based materials, a high-concentration two-dimensional electron gas (2DEG) channel can be formed at the AlGaN/GaN interface of GaN HEMT devices, significantly enhancing carrier mobility. Consequently, the theoretical figure of merit limit of GaN HEMT is higher than that of Si and SiC-based power electronic devices. In recent years, GaN HEMTs have been intensively studied and widely used in RF amplifiers and power electronics systems [3,4,5].

Despite the many benefits of GaN-based HEMT devices, the presence of traps in the bulk and at the interface can cause stability and reliability issues such as current collapse [6,7,8,9,10,11], threshold voltage drift [7,12], deterioration of short channel effect [12], and limited microwave power output [8,13], which seriously limit its large-scale applications. Therefore, trap characterization is crucial for achieving better commercial applications of GaN-based HEMT devices, which can provide more insights into the performances of devices as well as more guidelines for the optimization of the device structure and manufacturing processes to improve both the capability and reliability of GaN HEMTs. In recent decades, several techniques such as low-frequency noise (LFN), frequency dispersion properties, and deep-level transient spectroscopy (DLTS) have been developed to characterize the locations, types, concentrations, energy levels, and capture cross-sections of traps in GaN HEMTs, which are also applicable to novel GaN HEMT structures [14] and GaN diodes [15], p-n junctions [16], etc.

## 2. Types and Impacts of Traps

**Figure 1:**
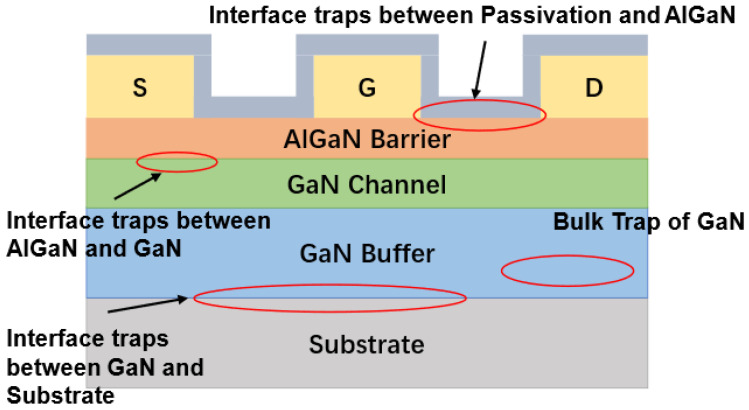
The locations of traps in GaN HEMTs.

Various traps found in GaN HEMTs are classified in this chapter. As illustrated in Figure 1, the main traps in GaN HEMTs can be classified into interface traps and bulk traps according to the locations; the former ones are located mainly between the AlGaN/passivation layer, GaN/Substrate, and AlGaN/GaN heterojunction, while the latter ones are located primarily in the GaN buffer layer and AlGaN barrier layer. In addition, for metal-insulator-semiconductor heterojunction field-effect transistors (MIS-HEMT), interface defects also exist at the interface between AlGaN and insulation.

The AlGaN/GaN interface and semiconductor/insulator interface traps are mainly caused by dislocations and defects generated during the growth of the material and the manufacturing processes of the device [17,18]. The reasons for the formation of bulk defects in the AlGaN barrier layer and GaN buffer layer are multifaceted. Firstly, buffer traps are introduced because of the high-resistance characteristics exhibited by the GaN buffer layer, which are usually achieved through C or Fe impurity compensation [19,20,21,22,23]. The 2DEG concentration of a device may be affected by the aforementioned traps [24], which, in turn, affects parameters such as current density and threshold voltage [25]. Secondly, *V*_Ga_-impurity, *V*_N_-impurity, Mg-H complexes, *V*_N_-Mg complexes, etc., also form point defects in GaN materials, introducing deep-level traps. In addition, although there has been significant development in GaN-on-GaN homoepitaxial growth and device fabrication [26,27], a considerable portion of devices still use heteroepitaxial substrates, in which large amounts of dislocations and defects are caused by lattice mismatch during the epitaxial growth process, forming deep-level trap states in the bandgap.

**Figure 2:**
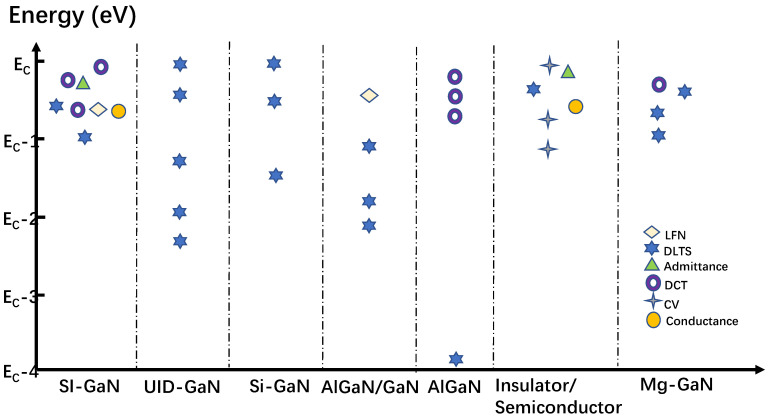
Energy levels and positions of traps in GaN HEMTs.

The energy levels, positions, and corresponding characterization methods of the traps identified in GaN HEMT devices are shown in Figure 2 [28,29,30,31,32,33,34,35,36,37,38,39,40,41,42,43,44,45,46,47,48,49,50,51,52,53,54,55,56,57,58,59,60,61,62,63,64,65,66]. From this figure, it can be observed that there are traps located near the energy band of 0.6 eV in SI-GaN/UID-GaN/Si-GaN/Mg GaN, which is said to be attributed mainly to the point defects in GaN in some papers, but there are other reports saying that the source of this type of trap may be caused by *V*_N_-impurity, Mg/Si-H complexes, and so on. The 0.7 eV trap near the AlGaN/GaN interface is generally believed to be caused by the spreading defects in the AlGaN/GaN heterostructure. And, beyond that, there are traps located around Ec-0.6 eV at the passivation layer and the semiconductor interface, which may be caused by the high Ga-O components near the passivation layer and semiconductor interface.

From the perspective of characterization methods, DCT, LFN, and low-frequency output admittance methods are used more frequently to characterize traps in semiconductor bodies, while other methods such as C-V and dispersion of conductance output are mainly used to characterize the traps at semiconductor interfaces. It is also illustrated that DLTS can characterize traps on the surface and in vivo based on their modes. In addition, it can be observed that DCT is usually used to characterize shallower traps, while DLTS can characterize deeper-level traps. DCT, a method that is used to characterize shallower traps, is different from DLTS since DLTS is usually used to characterize deeper traps.

From the perspective of trap location, DCT, DLTS, and C-V can identify the trap location through different voltage biases, and the conductivity method can measure semiconductor/insulator interface traps in MOS structures. As for LFN and transconductance methods, although they cannot identify trap locations, they can be used together with other characterization methods to comprehensively analyze traps.

## 3. Characterization Methods of Bulk Traps

### 3.1. Drain Current Transient

The drain current transient (DCT) test involves applying large positive *V*_ds_ bias, large negative *V*_gs_ bias, or both to measure the change in *I*_DS_ [48,49,50,51,52,53,54,55,56]. The transient current *I*_DS_ can be expressed as

(1) $$I_{\mathrm{DS}}\left(t\right)=\sum \Delta I_{i}\exp\left(-\frac{t}{\tau_{i}}\right)+I_{\infty },$$

where $\Delta I_{i}$ is the amplitude, $\tau_{i}$ is the time constant of the trap, and $I_{\infty }$ is the current which is at a steady state [51]. The Bayesian deconvolution method can be used to obtain the time constant of traps ($\tau_{n}$) and the energy level and cross-section of the trap can be derived from Arrhenius plots:

(2) $${\ln(\tau }_{n}T^{2})=-\frac{E_{a}}{k_{B}T}+\ln(\sigma_{n}\gamma_{n}),$$

where $\sigma_{n}$ is the electron capture cross-section, $\gamma_{n}$ contains the density and thermal velocity of electrons, $E_{a}$ is the trap activation energy, and $k_{B}$ is the Boltzmann constant [51].

The type of trap can be determined by the peak in the derivative spectrum of DCT where a positive peak represents the existence of an electron trap, while a negative peak shows that there exists a hole trap.

**Figure 3:**
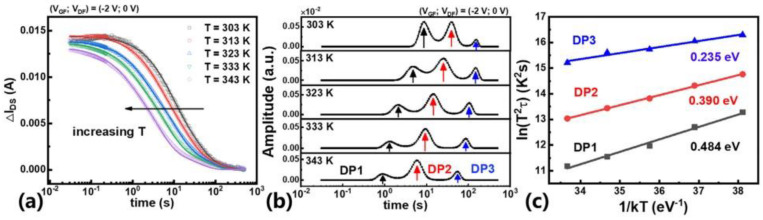
(Color online.) (**a**) The actual detrapping transient curves with different temperatures. (**b**) The time constant spectra at different temperatures. (**c**) The Arrhenius plots and the corresponding energy levels. Reprinted from [51], with the permission of AIP Publishing.

The process of extracting trap parameters with a leakage current response is illustrated in Figure 3. The change of *I*_DS_ at different temperatures during trap launch, the time constant of the trap extracted through the Bayesian convolution method, and the Arrhenius plot extracted from the transient curve are shown in Figure 3a, Figure 3b, and Figure 3c, respectively.

The physical location of the traps can still be determined by other means, although the inherent spatial sensitivity cannot be provided by this method. Different filling pulse conditions with different combinations of the gate-source voltage (*V*_gs_) and the gate-source voltage (*V*_ds_) stress conditions can be used to fill traps in different areas of GaN HEMTs. For instance, filling pulses with a strong negative *V*_gs_ < *V*_th_ can cause electrons to fill surface channel regions located beneath the gate area or defects in both the gate electrode and drain regions [67]. The channel capture can be highlighted by applying a strong positive *V*_ds_ bias and a strong negative *V*_gs_ bias due to the increase in the electron tunneling in the drain direction. A strong positive *V*_ds_ bias with *V*_gs_ > *V*_th_ can allow electrons to be captured in the barrier layer or buffer layer between the gate and drain, as large positive *V*_gs_ can scatter hot electrons out of the channel [68]. Therefore, the physical location of traps can be distinguished through DCT tests on a device performed by stress conditions.

The DCT technique is simple and can locate trap positions, but the trap density cannot be quantitatively measured and traps can only be detected with energy levels below 1 eV. Therefore, other approaches are necessary to characterize deep-level traps.

### 3.2. Low-Frequency Leakage Noise

**Figure 4:**
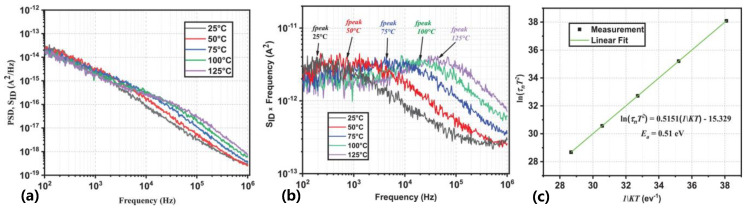
(Color online.) (**a**) Output noise PSD versus frequency. (**b**) Output noise PSD multiplied by frequency measured. (**c**) Extracted Arrhenius plot using LFN measurement [39].

Low-frequency leakage noise (LFN) is a noise signal generated in the low-frequency range, usually below a few hundred Hz, which reflects the charge and energy-level distribution inside a device. In GaN HEMT devices, the presence of traps affects the device’s leakage current and conductivity [69]. A small current noise is generated by traps when a small signal voltage is applied to the device. This noise can be measured by means of low-frequency leakage noise measurement techniques [39,56,60,61]. By analyzing the noise signals at different frequencies, parameter energy levels and capture cross-sections can be obtained by analyzing the noise signals at different frequencies and extracting the time constants of the G-R. The relationship between output noise and frequency is illustrated in Figure 4a. The measured output drain noise spectral density must be multiplied by the frequency to distinguish G-R noise from other measurement noise sources, as shown in Figure 4b. The cutoff frequency of traps can be extracted at different temperatures and then the trap parameters can be extracted by using the Arrhenius equation in Figure 4c.

Compared to DLTS and DCT, LFN has more difficulty in locating traps and lacks quantitative measurements of trap density, but it does not require a large reverse bias voltage to be applied to a device to degrade the de-trapping performance, and it is also capable of detecting traps in small area devices.

### 3.3. Low-Frequency Output Admittance Measurements

The characterization technique for low-frequency output admittance measurements characterizes traps by measuring the characteristics of their *S*/*Y* parameters as a function of frequency [39,57,58,59,65,70]. Then, the measured parameters can be calculated as equivalent *Y*_22_ parameters. Due to the influence of traps, the *Y*_22_ parameter obtained will reach its peak at a certain frequency. This peak will shift towards a higher frequency as the temperature increases. The emission time constant of the trap can be extracted from the peak frequency (*f*_peak_) using Equation (3). The parameters of the trap can then be obtained using the Arrhenius equation.

**Figure 5:**
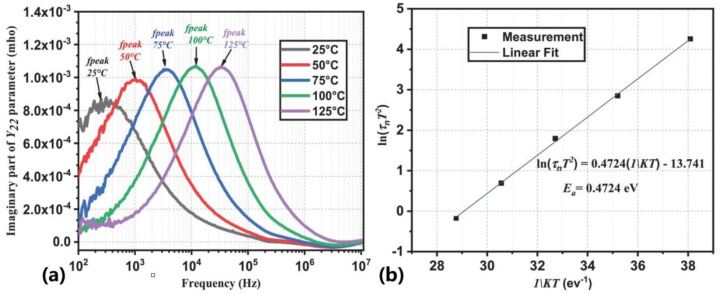
(Color online.) (**a**) Imaginary part of the measured *Y*_22_-parameter vs. frequency. (**b**) Extracted Arrhenius plot [39].

(3) fpeak=fImagY22=12πτn,

The relationship between the imaginary part of the measured *Y*_22_ parameter and the frequency is demonstrated in Figure 5a, and the Arrhenius plot of this measurement is shown in Figure 5b.

In practical applications, the appropriate parameter to represent the dispersion of HEMT traps should be chosen based on specific requirements. Generally, if an analysis of the influence of HEMT traps on the entire system is required, the S parameter should be used as it provides the relative response between input and output ports. On the other hand, if a deeper understanding of the characteristics of HEMT traps is required, the Y parameter may be more suitable as it provides the internal response of the device, including the relationship between voltage and current.

Pulse effects such as voltage stabilization time or unstable temperature are avoided and wide dynamic range and measurement speed are provided in this method [70]. However, only energy levels and capture cross-sections for traps can be obtained through this method.

### 3.4. DLTS

**Figure 6:**
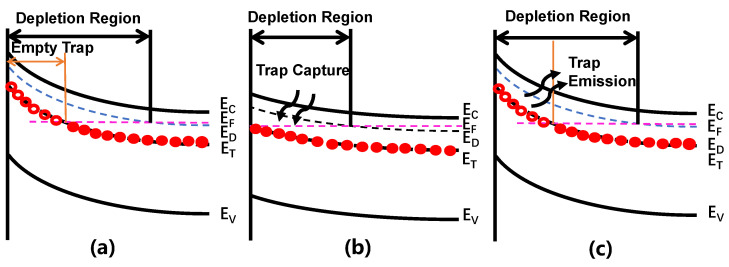
(Color online.) Schottky diode energy-band diagrams: (**a**) steady-state; (**b**) trap filling state; (**c**) trap emission state.

DLTS has the advantages of being sensitive to measurement, having a wide range of detectable defect energy levels, being able to simultaneously measure both majority and minority carrier traps, and being able to determine trap positions. The processes involved in trap emission and capture during DLTS testing are summarized in Figure 6. In the steady-state condition, as indicated in Figure 6a, the energy level *E*_T_ is not occupied by electrons. The Fermi level is forced to shift towards the conduction band when applying a filling pulse bias voltage (*V*_f_), as indicated in Figure 6b, which attracts electrons and consequently weakens the built-in electric field. The trap levels within the depletion region are situated below the Fermi level. Charges captured by traps within the depletion region with energy levels above the Fermi level will be emitted by applying a measurement voltage (*V*_m_) that is more negative than the filling pulse voltage *V*_f_, as shown in Figure 6c. Taking the contribution of thermal emission into account, the trap emission constant can be obtained by measuring the change in capacitance through the DLTS from time *t*_1_ to *t*_2_ after the pulse [71]. As for the determination of the type of trap, the method of identifying the type of peak in the derivative of capacitance versus the time plot can be applied, where a positive peak represents the trap type as an electron and a negative peak represents the trap type as a hole. Taking an n-type semiconductor as an example, the trap density can be calculated from Equation (4).

(4) $$N_{T}=\frac{∆C_{max}}{C_{0}}\frac{2N_{D}r^{\frac{r}{r-1}}}{r-1},$$

where r = *t*_2_/*t*_1_ and $C_{0}$ is the steady-state capacitance value.

Capacitor DLTS (C-DLTS) first applies a filling pulse voltage to the device being tested and then measures the change in capacitance after the pulse to characterize the trap [35,72,73,74,75]. Traps in the AlGaN barrier layer, GaN channel layer, and buffer layer can be effectively distinguished through C-DLTS. The main principle is based on the state of the 2DEG whereby the gate capacitance primarily comes from the GaN channel layer and buffer layer, while the contribution of the barrier layer to the total capacitance is very small when the 2DEG is depleted, and the depletion region is mainly confined to the AlGaN barrier layer when the 2DEG is accumulated.

It should be noted that the transient values of C-DLTS are usually less than 10% of the total depletion capacitance. Therefore, a sufficient area must be produced to enable experimental resolution ΔC. Furthermore, it is necessary to consider the signal-to-noise ratio of DLTS devices.

**Figure 7:**
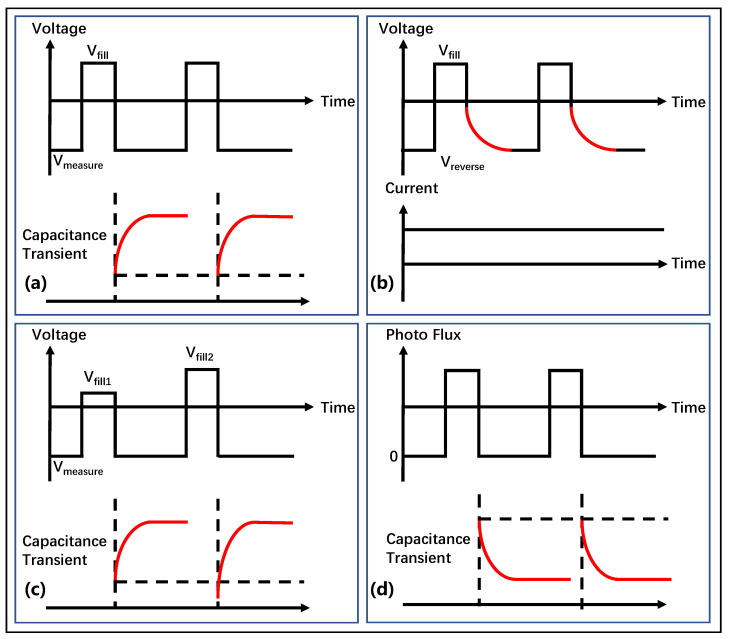
(Color online.) Measurement conditions and curves for (**a**) C-DLTS, (**b**) CID-DLTS, (**c**) D-DLTS, and (**d**) O-DLTS.

Constant drain-current DLTS (CID-DLTS) is applied to obtain specific trap parameters beneath the gate by adjusting the *V*_GS_ to maintain a constant drain current (*I*_DS_), as depicted in Figure 7b. In the gate-controlled mode, the gate voltage is pulsed to *V*_fill_ in order to populate the deep levels, and the *I*_DS_ must be kept constant in order to measure the transient response of *V*_GS_. The merits of CID-DLTS are that it can detect traps at very low concentrations and capture trap responses with high sensitivity as well as in very short time scales within the device [37,46].

Double correlation DLTS (D-DLTS) replaces one amplitude pulse in C-DLTS by using pulses with different amplitudes, as shown in Figure 7c. Although this method makes the experiment and data processing more complex, it facilitates the observation of defect behavior within the space–charge region. Moreover, the variation of defects with depth can be analyzed by changing the pulse amplitude and rate window [76,77].

In DLTS technology, there is also a commonly used drain current DLTS (I-DLTS), whose testing method is highly similar to that of DCT technology, except that DCT observes long-term changes in *I*_DS_, while I-DLTS selects a small interval with significant current changes for analysis.

Several of the above DLTS techniques are suitable for detecting majority traps, while the detection of minority traps is difficult. This is due to the very stringent test conditions for minority traps, which require a few traps to be filled and majority traps to be emptied. The optical DLTS (ODLTS) technique has been proposed and validated in order to better characterize minority traps.

ODLTS is a method that uses light pulses as injection pulses to excite electrons and holes, causing carriers to be captured from the trap. Once the light pulse is switched off, the carriers are detrapped so that the capacitance of the device can be measured to obtain the trap parameters. As shown in Figure 7d, photo-generated carriers are generated and captured by traps when a light pulse is applied to a device. After the end of the optical pulse, these captured carriers are de-captured, causing a gradual change in capacitance. Trap parameters can be extracted from capacitance changes at different temperatures after going through the above steps. One of the superiorities of ODLTS is that it overcomes the limitations of DLTS in studying minority traps, with high sensitivity to ultra-deep-level traps [42].

So far, the conventional DLTS method described has the drawback of poor energy resolution. Laplace DLTS (L-DLTS) is an isothermal technique in which the capacitance transients at a fixed temperature are digitized and averaged, and then the defect emission rate is obtained through numerical methods equivalent to the Laplace inverse transform [78]. Compared with traditional DLTS, the main advantage of L-DLTS is a significant improvement in energy resolution, which provides a more precise detection for traps. However, a better signal-to-noise ratio is required in L-DLTS than in conventional DLTS, which makes it less sensitive by a factor of 5.

## 4. Characterization Methods of Interface Traps

### 4.1. Constant Capacitance Deep-Level Transient Spectroscopy

**Figure 8:**
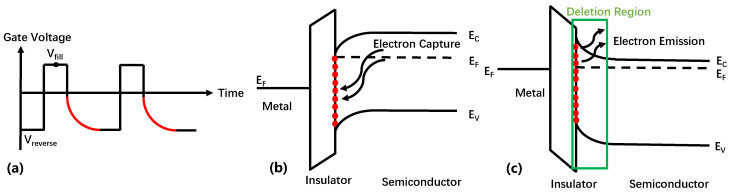
(Color online.) (**a**) The process of CC-DLTS measuring MIS HEMT interface traps. Energy-band diagrams for an MIS HEMT on an n-type semiconductor for (**b**) pulsed accumulation bias and (**c**) nonequilibrium depletion bias.

Constant capacitance DLTS (CC-DLTS) employs a customized feedback control circuit to maintain a consistent capacitance and regulate voltage to measure voltage transients resulting from trap discharge [66,79,80]. Significant advantages of this method over the traditional DLTS are evident as it ensures a constant depletion of capacitance and SCR width throughout the entire transient process. This feature is particularly beneficial when studying the interface traps in MIS-HEMTs since it keeps the Fermi level constant during the transient response process. Figure 8 illustrates the process and principle of CC-DLTS for measuring MIS HEMT interface traps.

As shown in Figure 8a, the MIS-HEMT is biased at a negative pressure *V*_reverse_, and a depletion capacitor is established at *V*_reverse_. Following that, a pulsed filling voltage referred to as *V*_fill_ is employed to transition the device into an accumulation state. Throughout this phase, electrons are introduced and occupy the interface/oxide states, as demonstrated in Figure 8b. After the filling pulse, the device returns to the depletion state, and the traps emit electrons. The depletion bias is used to maintain a constant capacitance, which means maintaining an almost-fixed SCR. At this condition, the change in bias voltage is reflected by the release process of interface traps, and the energy and density of the traps can be extracted from *V*_ds_(t). Equation (5) allows for the calculation of the interface state density (*N*_it_).

(5) $$N_{\mathrm{it}}=\int_{E_{\mathrm{FR}}}^{E_{\mathrm{FP}}}N_{\mathrm{SS}}(E)\;(1-\exp(\frac{{-t}_{P}}{\tau_{C}\left(E\right)}))\mathrm{dE},$$

It involves the density of detected interface states ($N_{\mathrm{SS}}\left(E\right)$), the Fermi level at the gate bias of UR ($E_{\mathrm{FP}}$), the Fermi level at the gate bias of UP ($E_{\mathrm{FR}}$), and the capture time constants ($\tau_{C}\left(E\right)$) associated with the interface states [79].

Nevertheless, CC-DLTS has a slow response due to the influence of the feedback circuit, but it is still highly sensitive and suitable for measuring interface traps [81,82,83,84].

### 4.2. Quasi-Static C-V Measurement

Quasi-static C-V (QSCV) testing is the process of testing the C-V curve of a device under quasi-static and high-frequency conditions. The C-V curve obtained at high frequencies is generally considered to be the ideal curve without interface traps, whereas the C-V curve changes in response to interface traps at low frequencies. The density of interface traps can be determined by comparing the C-V curves measured at quasi-static and high frequencies (HFCV) using Equation (6) [85,86].

(6) $$D_{\mathrm{it}}=\int_{V<V_{\mathrm{th}}}^{V_{G}}\left(C_{\mathrm{QSCV}}-C_{\mathrm{HFCV}}\right)dV/e,$$

**Figure 9:**
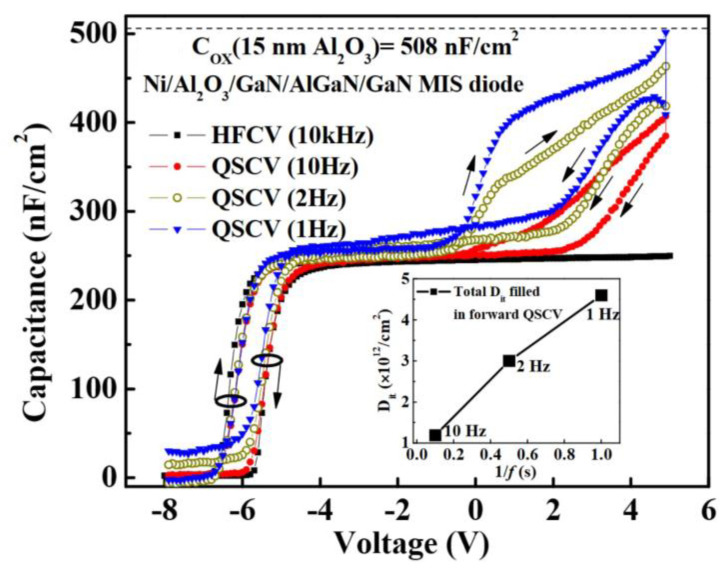
(Color online.) CV characteristics measured at 10, 2, 1, and 10 kHz of Ni/Al_2_O_3_/GaN/AlGaN/GaN MIS diodes. Reproduced with permission from [85]. Copyright 2012, Wiley.

Figure 9 demonstrates the CV testing of MIS diodes, with 10 kHz selected as the high frequency and 1 Hz as the lowest quasi-static state.

Quasi-static C-V measurement can provide not only the interface trap charge density but also the determination of the trap energy level and capture cross-section.

### 4.3. Dispersion of Conductance Output

The dispersion of conductance output is a highly sensitive technique for characterizing interface defect density, which is capable of detecting interface defects on orders of 10^10^ cm^−2^/eV or lower [63,64,87].

**Figure 10:**
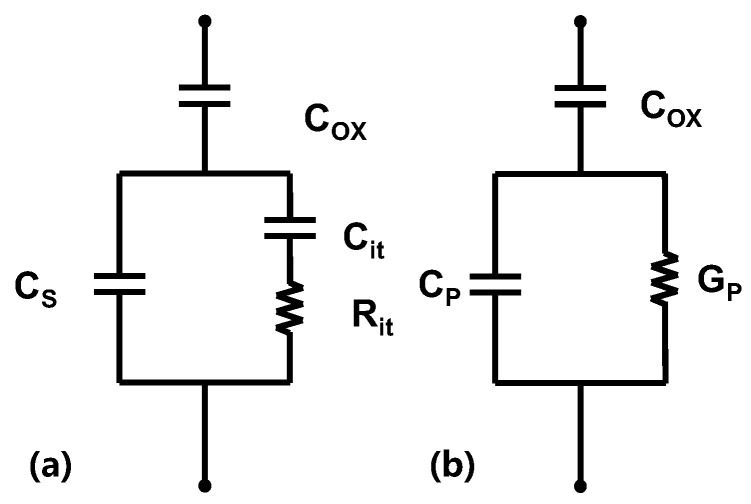
(**a**) The equivalent circuit of MOS. (**b**) Simplified circuit of (**a**).

The equivalent circuit for measuring MOS interface traps using the dispersion of conductance output method is shown in Figure 10a, where *C*_OX_ is the oxide layer capacitance, *C*_S_ is the semiconductor capacitance, and *C*_it_ is the interface trap capacitance. The charge loss caused by the trap capture emission is represented by *R*_it_. By circuit transformation, Figure 10b can be obtained, where *C*_P_ and *G*_P_ are represented by the Equations (7) and (8):

(7) $$C_{P}=C_{S}+\frac{C_{\mathrm{it}}}{1+{\left(\omega \tau_{\mathrm{it}}\right)}^{2}},$$

(8) $$\frac{G_{P}}{\omega }=\frac{qD_{\mathrm{it}}}{2\omega \tau_{\mathrm{it}}}\ln\left[1+{\left(\omega \tau_{\mathrm{it}}\right)}^{2}\right],$$

Here, $\tau_{\mathrm{it}}=C_{\mathrm{it}}R_{\mathrm{it}}$. By plotting the *G*_P_/*ω*-*ω* curve, the trap density $D_{\mathrm{it}}$ and the trap time constant can be extracted from the peak of the curve and the frequency corresponding to the peak, respectively.

The conductivity method is highly sensitive, but requires operation over a wide frequency range and is therefore slow to measure [87].

### 4.4. Single-Pulse Charge Pump

To characterize the interface trap density, a technique known as single-pulse charge pump (SPCP), a method of applying a pulse voltage to gates, can be applied on devices. The SPCP measurement process involves applying a high enough pulse to the gate when the gate voltage is small [88,89]. As the rising edge of the pulse advances, channel electrons begin to accumulate and, subsequently, a portion is captured by the interface trap. At the beginning of the falling edge, the trapped electrons cannot respond quickly due to the long trap time constant. As a result, a difference in current between rise time and fall time occurs, which determines the interface trap density, as shown in Equations (9) and (10):

(9) $$Q_{\mathrm{it}}=\int (I_{\mathrm{tcp},\mathrm{RISE}}-I_{\mathrm{tcp},\mathrm{FALL}})\mathrm{dt},$$

(10) $$Q_{\mathrm{it}}={qN}_{\mathrm{it}},$$

where $I_{\mathrm{tcp},\mathrm{RISE}}$ and $I_{\mathrm{tcp},\mathrm{FALL}}$ are CP current during rise time and fall time, respectively.

SPCP is less susceptible to interference from gate leakage and well characterized for traps with recovery times within 100 μs [89]. However, except for the value of the interface trap density, other parameters cannot be determined.

## 5. Conclusions and Outlook

The characterization of GaN HEMT traps is a complex and challenging task that requires advanced experimental techniques and sophisticated theoretical models. Although considerable advancements have been made in this domain, numerous unanswered questions and technical challenges remain, necessitating further exploration to comprehensively understand the characteristics and dynamics of these traps.

A significant obstacle arises from the restrictions imposed by existing measurement techniques. This review focuses on the principles and processes of characterizing traps using electrical, optical, and junction capacitance methods, which have already been widely used to probe traps in GaN HEMTs. However, these techniques have their own drawbacks, such as low sensitivity, poor spatial resolution, and limited frequency range. Furthermore, some of these techniques require special sample preparations or expensive equipment, which can limit their accessibility and practicality. The scope and characteristics of each characterization technique are summarized in Table 1.

Another challenge is related to the characterization of interface traps. The characterization of GaN HEMT interface traps is mostly applicable to MIS HEMT, while the interface traps of conventional HEMT structures are difficult to measure due to the presence of MS junctions.

To overcome these challenges, further optimizations and innovations in characterization techniques are needed. The characterization of GaN HEMT traps will continue to be a vibrant field, with many exciting opportunities, such as the new measurement techniques that can provide higher sensitivity and resolution a and wider frequency range while reducing the cost and complexity of further research and developments. Moreover, by integrating multiple measurement techniques and theoretical models, complementary and consistent information about traps can be obtained.

In conclusion, the characterization of GaN HEMT traps is a challenging and rewarding task that is critical for improving device performance and reliability. With continuous advancements in measurement techniques and theoretical models, it can be anticipated that profound understandings of the characteristics and dynamics of these traps can be further unlocked so that the full potential of traps for future applications can be realized. This knowledge will enable us to enhance the stability and reliability of GaN HEMTs, broaden their range of applications, and unleash their complete potential for future utilization.

## Figures and Tables

**Table 1:** The range of applications and characteristics of characterization methods.

Methods	Range of Application	Sensitivity	Speed	Non-Destructiveness	Characteristics
DCT	Bulk trap	High	Low	No	Easily interferes with leakage current
LFN	Bulk trap	High	Fast	Yes	Easily interferes with noise
Low-frequency output admittance	Bulk trap	Low	Fast	Yes	Wide range
C-DLTS	Bulk trap	High	Low	No	Wide range
CID-DLTS	Bulk trap	High	Low	No	Complex
D-DLTS	Bulk trap in the SCR	High	Low	No	Complex
ODLTS	Bulk trap	High	Low	No	Measures minority carrier traps
CC-DLTS	Interface trap with high concentrations	High	Low	No	Complex
QSCV	Interface trap with high concentrations	Low	Fast	Yes	Energy levels and capture cross-sections cannot be obtained
Dispersion of conductance	Interface trap	Most high	Low	Yes	Wide frequency range
SPCP	Interface trap	High	Fast	Yes	Energy levels and cross-sections unknown

## Data Availability

Data sharing not applicable.
